# Supplementary material for: Field-based tree mortality constraint reduces estimates of model-projected forest carbon sinks
Source: Nat Commun. 2022 Apr 19;13:2094. doi: 10.1038/s41467-022-29619-4 (PMC9018757; doi:10.1038/s41467-022-29619-4)
Supplement: Supplementary file 1 — Supplementary Information [file 41467_2022_29619_MOESM1_ESM.pdf]

Supplementary Information for

Field-based tree mortality constraint reduces estimates of model-projected forest carbon sinks

Kailiang Yu[1,2*], Philippe Ciais[1], Sonia I. Seneviratne[3], Zhihua Liu[2], Han Y.H. Chen[4], Jonathan Barichivich[1,5], Craig D. Allen[6], Hui Yang[1,7], Yuanyuan Huang[1,8], Ashley P. Ballantyne[1,2]

[1]Le Laboratoire des Sciences du Climat et de l'Environnement, IPSL-LSCECEA/CNRS/UVSQ Saclay, Gif-sur-Yvette, France

[2]Department of Ecosystem and Conservation Sciences, University of Montana, Missoula, USA

[3]Institute for Atmospheric and Climate Science, ETH Zürich, Zürich, Switzerland

[4]Faculty of Natural Resources Management, Lakehead University, Thunder Bay, Ontario, Canada

[5]Instituto de Geografía, Pontificia Universidad Católica de Valparaíso, Valparaíso, Chile

[6]Department of Geography and Environmental Studies, University of New Mexico, Albuquerque, NM, USA

[7]Max Planck Institute for Biogeochemistry, Jena, Germany

[8]CSIRO Oceans and Atmosphere, Aspendale, Australia

Corresponding author: Kailiang Yu, kai86liang@gmail.com

Forest plot datasets

We compiled forest inventory datasets through a comprehensive review of literature and long-term forest inventory datasets. The majority of forest plot datasets in North America were derived from Canada forest plots and the Forest Inventory and Analysis (FIA) Program of the U.S. Forest Service. We here provided a summary for these plots in Canada and USA. In datasets in Canada, only natural forests plots were used and forest age with more than 80 years were used to select the mature or old-growth forests. These plots are located in Alberta (AB), Saskatchewan (SK), Manitoba (MB), Ontario (ON) and Quebec (QC). These datasets have been used in previous studies to examine forest growth and mortality[1–3]. Thus, the readers could refer to these studies for details of plot design, establishment, and vegetation survey. As briefly summarized here, these plots were established in visually homogenous well-stocked stands and were located at least 100 m from any openings to minimize the impacts of edge effects. All trees within the plot that met the diameter threshold were tagged and their species names were recorded. Canada forest plots have good records of vegetation regeneration history and thus allowed us to select the natural regenerated forests stands that were largely unmanaged. For the purpose of this study, we used the available data of aboveground woody biomass loss (LOSS) from mortality. Ultimately, the 1803 mature nature forest plots without large disturbance ranging from 1951 to 2016 were screened from more than 4000 forests plot to meet the criteria stated in the main text.

In FIA datasets, only natural forests plots were used and percent of harvest <10% were further used to select the plots with minimal human disturbance. We note that the majority of forests stands have been forest recovery or regrowth following past disturbance in FIA datasets and they are thus young stands[4,5], while there are a large number of forest plots (i.e., n > 13000). Consistent with previous studies[6], forest age with more than 100 years were used to select the

mature or old-growth forests, while we note that forest age was estimated as the average of three dominant or co-dominant trees that represent a plurality of non-overtopped trees. All trees (standing live and dead), with a diameter at breast height (DBH) of at least 12.7 cm, are inventoried in each plot. It is worthy of clarifying that the criteria of 12.7 cm is much higher than Canada forests. Thus, FIA forest plot datasets could underestimate growth because of limited records of recruitments. This further demonstrates the advantage of using biomass loss (LOSS) from mortality as observations to constrain forest carbon sink in this study. Finally we used 242 mature forest plots ranging from 1997 to 2018 in FIA datasets to meet the criteria stated in the main text. The rest forest plots datasets in North America were derived from publicly available long term datasets of LOSS in Western USA (n = 58 ranging from 1955 to 2011) and Alaska (n = 112 ranging from 1995 to 2014)[6]. In Alaska plot datasets, the mature forests were judged by forest gymnosperm fraction[7].

The mature forest plot datasets in tropical regions were mainly derived from three literatures with original data sources from RAINFOR, AfriTron, and ForestGEO[8–10]. The readers could refer to these studies for details of plot design, establishment, and vegetation survey. Here we provided a summary of these plots. The study by Brienen et al (2015)[8] examined the forest growth and mortality and forest carbon sink in Amazon and they compiled data of total 321 plots. Yu et al (2019)[6] further augmented this dataset with adding the forest plots derived from Smithsonian Tropical Research Institute (11 plots, called ForestGEO - STRI plots) and CARBONO project conducted in La Selva biological station (18 plots, called CARBONO plots) to study the temporal changes in carbon turnover time. To meet the criteria stated in the main text, we finally used 212 forest plots ranging from 1975 to 2014 in Amazon. The study by Hubau et al (2020)[9] further used the long term datasets in Africa plus the forest datasest in Amazon to

compare the growth, mortality and carbon sink in African and Amazonian tropical forests. The study by Sullivan et al (2020)[10] augmented these datasets by adding forest plots in Asia & Australia to study the long-term thermal sensitivity of earth's tropical forests. In our study, we used the available data of aboveground biomass loss from mortality (Mg ha^{-1} y^{-1}) from these tropical plots and ultimately used 249 plots ranging from 1964 to 2016 in Africa and Asia & Australia. In all of these datasets, the publicly available aboveground biomass loss from mortality (Mg ha^{-1} y^{-1}) was quantified through tree mortality in each census interval, following the methods reported in these previous studies. Supplementary Table 3 provided a summary of the number, size, time range of these forest plots used in this study across continents – North America, South America (Amazon), Africa, Asia & Australia. The readers could also refer to other potential sources of data such as Global Forest Carbon Database (ForC)[11] https://forc-db.github.io/ to augment the database used in this study.

Geospatial modeling and environmental drivers

We used machine learning (random forest) to generate a map of woody biomass loss from mortality (LOSS) across continents. The long-term forest plots were treated as distinct samples (n = 2676). All sample points falling within the same 0.25 degree grid were averagely aggregated, which lead to a total of 814 unique grids across continents as inputs into the models. A stack of 57 ecologically relevant, global map layers including climatic, soil nutrient, soil chemical, soil physical, vegetative indices, radiation and topographic variables and one anthropogenic covariate (Supplementary Data 1) were used to determine the independent variables which could affect LOSS. All of these covariate map layers were standardized at 0.25 degree. If layers have a higher original grid resolution, these layers were downsampled using a mean aggregation method. When layers have a lower original resolution, these layers were

resampled using simple upsampling (i.e., without interpolation) to align with the higher resolution grid. Each sample (plot)-specific independent variables were then derived from the 57 ecologically relevant, global map layers based on each sample' georeferenced location.

Geospatial modelling was used to investigate the dependence of LOSS on the 57 covariates. Random forest with a variety of parameters (i.e., variablesPerSplit 2, 3, 4, 5, 8, 10) to train the models and assessed each model using 10-fold cross validation[12]. The coefficient of determination values for each fold of data were used to compute mean and standard deviation values for the cross validated model. The best model with highest coefficient of determination values and lowest standard deviation was finally used to spatially extrapolate the LOSS across continents. The results showed that the best performing model had high predictive strength (overall $R^2 = 0.93$).

Mask of natural forests

To generate the map of LOSS in natural forests, we used rasters of tree cover, human footprint index, percent annual burn area and managed land cover as mask to define the natural forest areas. Advanced Very High Resolution Radiometer (AVHRR) Continuous Fields Tree Cover Product (1 kilometer)[13] with more than 10% was used to define forests. The criteria used to define natural forests with minimal disturbance include: Human Footprint Index - HFI <50%, percent annual burn area averaged from 1996 to 2015 < 30%, and cultivated and managed vegetation cover < 30%. The Global Human Footprint Dataset (HFI) (1 kilometer) of the Last of the Wild 220 Project, Version 2, 2005 (LWP-2) expressed as a percentage was used to account for human activity and is from http://sedac.ciesin.columbia.edu/data/set/wildareas-v2- 222 human-footprint-geographic. Data of GFED4 biomass burning emissions (0.25° × 0.25°) expressed as percent annual burn area were used to account for the potential impacts of fires and were from https://daac.ornl.gov/VEGETATION/guides/fire_emissions_v4.html. Cultivated and managed

vegetation cover was derived from Global 1-km Consensus Land Cover product[14]. These masks were used at 0.25 degree in generating observational maps of LOSS and were used at 0.5 degree in other cases such as data-model comparison and emergent constraint.

Supplementary Table 1. Modeled projected (period 2015-2099, units in Pg C y^{-1}) grid-level NPP and grid-level HR before and after constrain across continent in six DGVMs

Continent	Before constraint		After constraint	
NPP	Mean	SD	Mean	SD
North America	6.1	2.9	6.2	2.6
South America	9.0	3.0	8.4***	2.3
Africa	2.4	0.8	2.3*	0.7
Asia & Australia	2.3	0.9	2.2***	0.6
HR				
North America	5.4	2.0	5.4	1.5
South America	7.0	1.7	6.5***	0.8
Africa	1.9	0.5	1.9	0.3
Asia & Australia	1.8	0.5	1.7***	0.2

Note: 1) the scenario is with accounting for the effects of projected climate – precipitation and temperature;

2) a bootstrapping (100 times) approach was used for constraint of projected NPP or HR (see Methods) and ensemble means were reported;

3) the ANOVA test was used to explicitly evaluate whether the difference before and after constrain of projected NPP or HR was significant. * for $P < 0.05$; ** for $P < 0.01$; *** for $P < 0.001$.

Supplementary Table 2. Mortality components used and not used in this study in DGVMs

DGVMs	Mortality components used in this study	Mortality components not used in this study
ORCHIDEE	Turnover due to mortality	Mortality due to fire
CABLE-POP	Mortality due to crowding, resource limitation	Mortality due to disturbance
JULES	Background mortality; Mortality due to competition	
LPJ-GUESS	Background mortality; Mortality due to low growth efficiency, negative biomass, plant allometry outside defined bounds and bioclimatic limits	Mortality due to fire and patch-destroying disturbance
LPJmL	Mortality due to low growth efficiency, competition, negative allocation and biomass, heat stress and reproduction	Mortality due to fire
SEIB-DGVM	Background mortality; Mortality due to low growth efficiency, heat stress and bioclimatic limits	Mortality due to fire and disturbance

Note: [1]The name of the components of mortality is based on the outputs in DGVMs.

[2]The type of disturbances in CABLE-POP and SEIB-DGVMs were not explicitly stated.

Supplementary Table 3. Summary of the compiled long-term (> 9 years) forest monitoring plot dataset ranging from 1951 to 2018 over at least three censuses across continents.

Continents	Number of plots	Total area (Ha)	Earliest census (y)	Latest census (y)	Data source or providers
North America	2215	274	1951	2018	Luo & Chen (2013, 2015)[1,2]; Chen et al (2016)[3]; Yu et al (2019)[6] ; FIA datasets
South America	212	360	1975	2014	Brienen et al (2015)[8]; Yu et al (2019)[6]
Africa	186	216	1968	2016	Hubau et al (2020)[9]; Sullivan et al (2020)[10]
Asia & Australia	63	51	1964	2015	Sullivan et al (2020)[10]

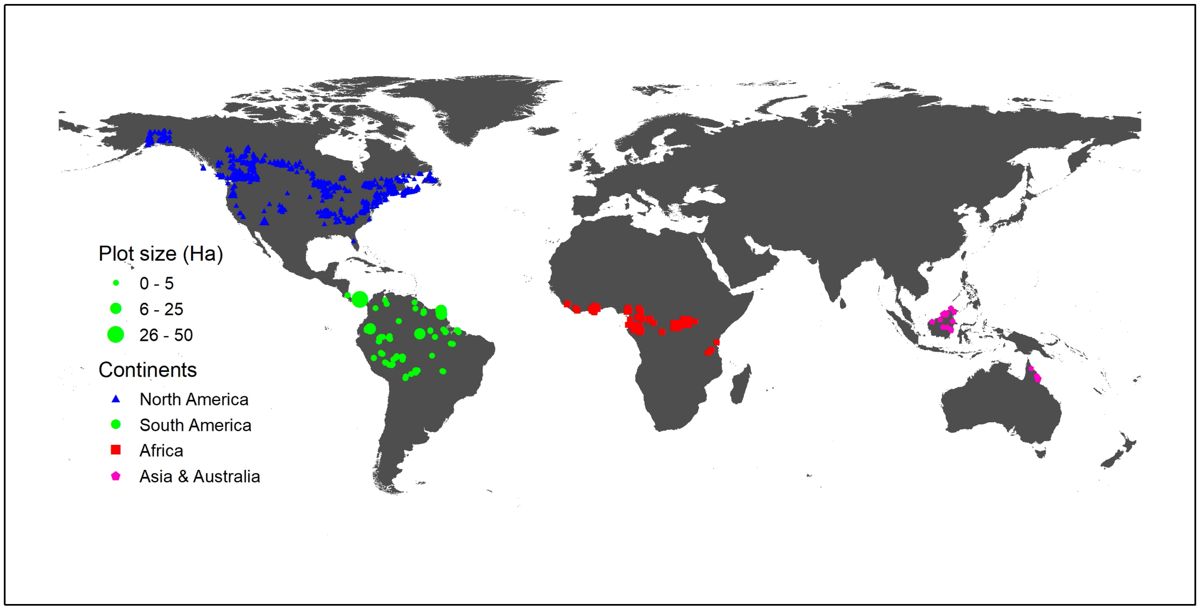

Supplementary Fig. 1. Distribution of largely unmanaged long term forest plot datasets (n = 2676) ranging from 1951 to 2018 across continents – North America, South America, Africa, and Asia & Australia.

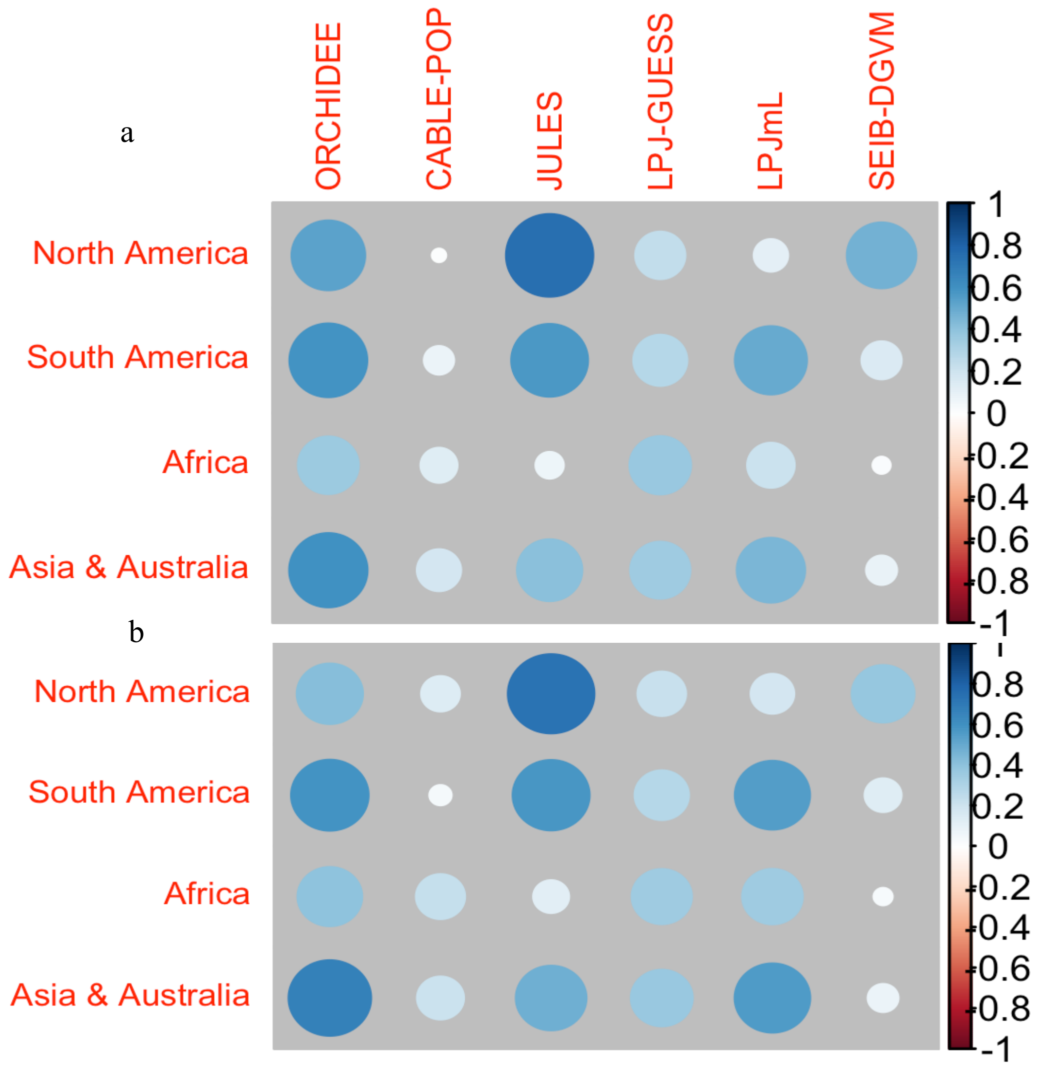

Supplementary Fig. 2. Standardized response coefficients between historic (1961-2014) and projected (2015-2099) grid-level NPP (a) and grid-level heterotrophic respiration (b, HR) across continents predicted by six DGVMs (ORCHIDEE, CABLE-POP, JULES, LPJ-GUESS, LPJmL, and SEIB-DGVM). All grids values were used within each continent.

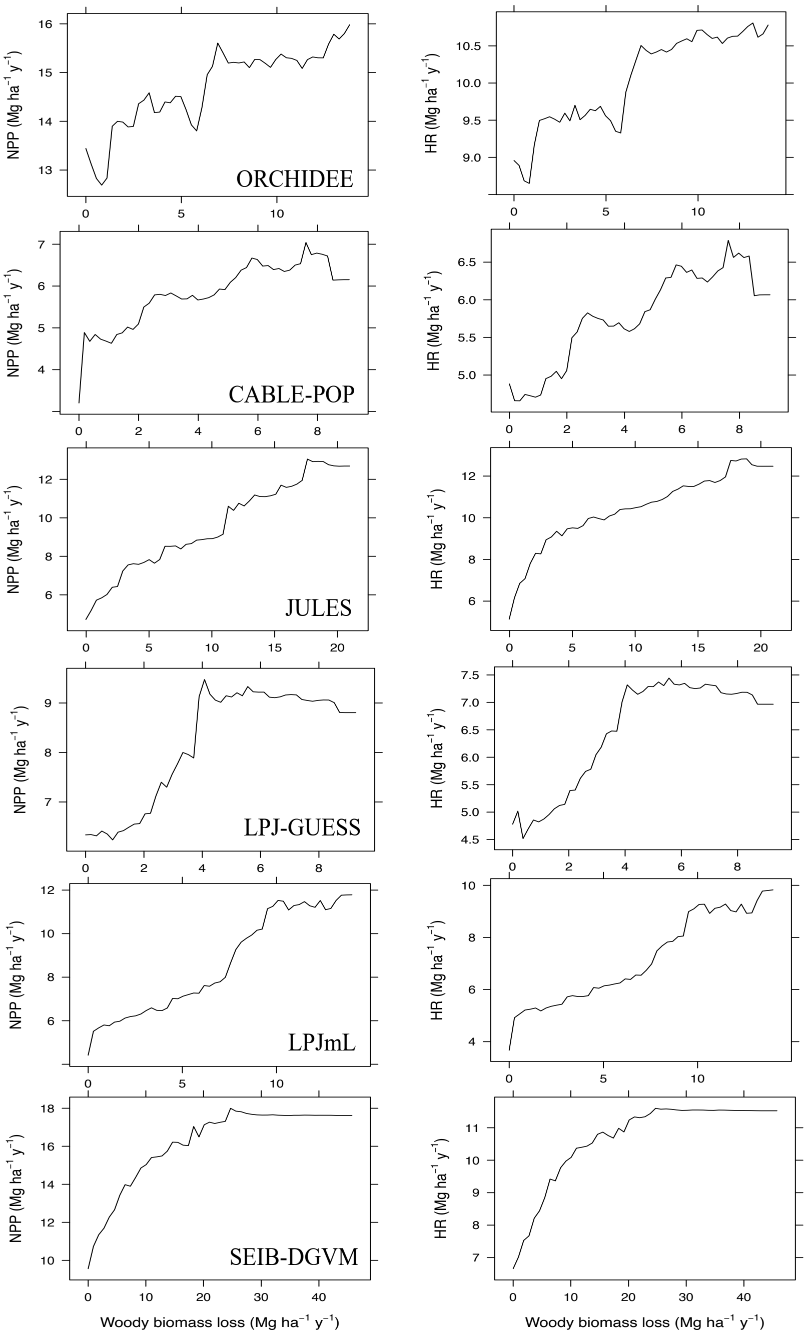

Supplementary Fig. 3. The relationships between historic (1961-2014) woody biomass loss from mortality and projected grid-level NPP and grid-level heterotrophic respiration (HR) predicted by partial feature contribution analysis of controlling mean annual temperature and mean annual precipitation and six DGVMs (ORCHIDEE, CABLE-POP, JULES, LPJ-GUESS, LPJmL, and SEIB-DGVM).

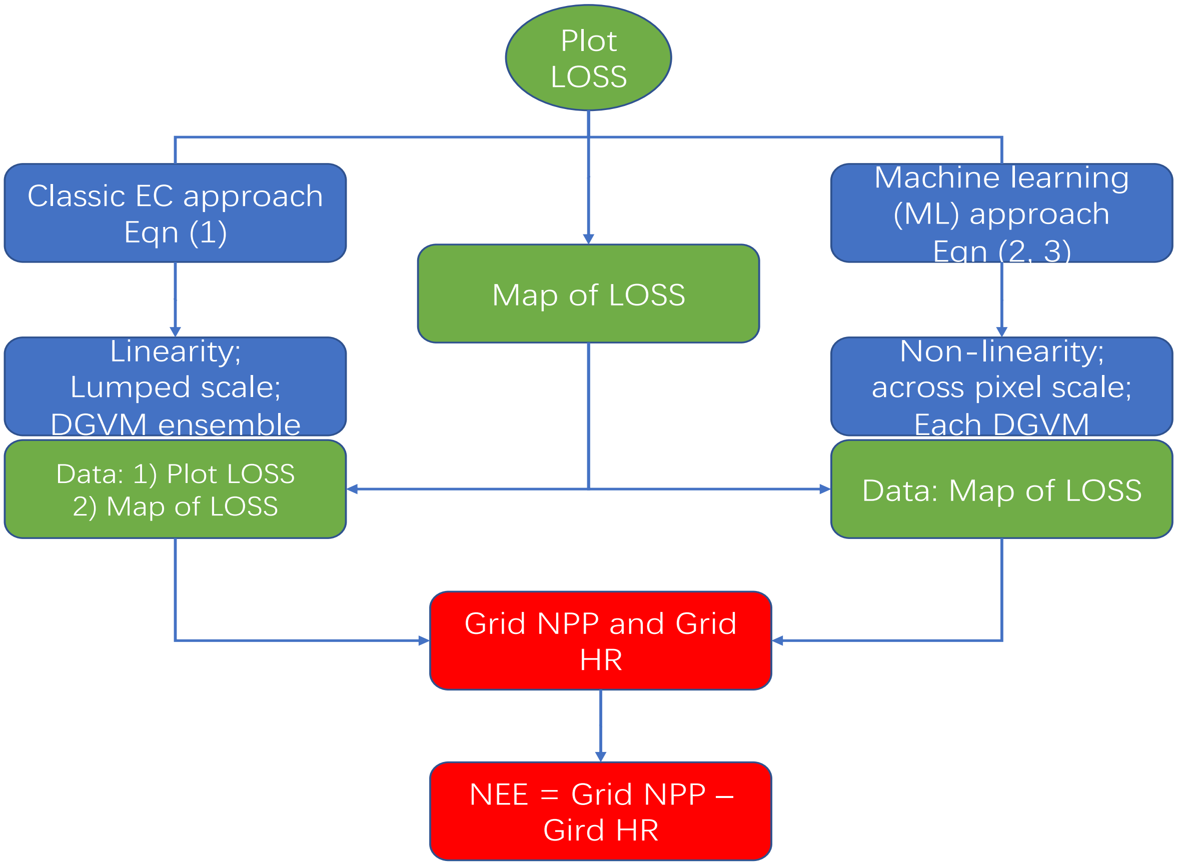

Supplementary Fig. 4. Schematic illustration of two approaches to constrain the projected forest carbon sink across continents. The conventional emergent constrain (EC) approach was achieved by identifying the statistic (linear) relationship between historical LOSS and projected NPP and HR within each continent – North America, South America, Africa, and Asia & Australia across DGVM ensembles. The conventional EC approach was implemented at lumped spatial scale[15,16] in which LOSS corresponding to local forest plot sites or within each continent at continental scale was aggregated as averages, and NPP and HR were aggregated as sums within each continent at continental scale for building their emergent linear relationships across DGVM ensembles. This thus essentially led to substantial reduction of sample size during constraint with lack of capability of using all values of LOSS either from original forest plots data or upscaled gridded maps of LOSS. In this sense, the data used to impose the constraint of NPP and HR in

conventional EC approach were derived from local forest plot original data or maps of LOSS using mean ± sd within each continent[15,16]. See examples of the Supplementary Fig. 11 and Fig. 12 for details. By comparison, the machine learning (ML) approach was used to examine the non-linear relationships by training a ML (random forest) model between historical LOSS and projected NPP and HR in each DGVM. The ML approach was conducted across grid scale in which all grid values of LOSS, NPP and HR were used to build their non-linear relationships in each DGVM. In the ML approach, maps of LOSS were used to feed into the trained ML (random forest) model to assess the impacts of historic LOSS on the projected NPP and HR. This ML approach thus allows for a spatially explicit constraint of projected NPP and HR in each model which can be sequentially aggregated at broad spatial scales. Maps of LOSS were generated by a ML (random forest) approach that linked spatial variations of LOSS at local-plot scale in a quasi-steady state (see Methods) with 57 environmental variables. Forest carbon sink was quantified by the difference of grid-level NPP and grid-level HR.

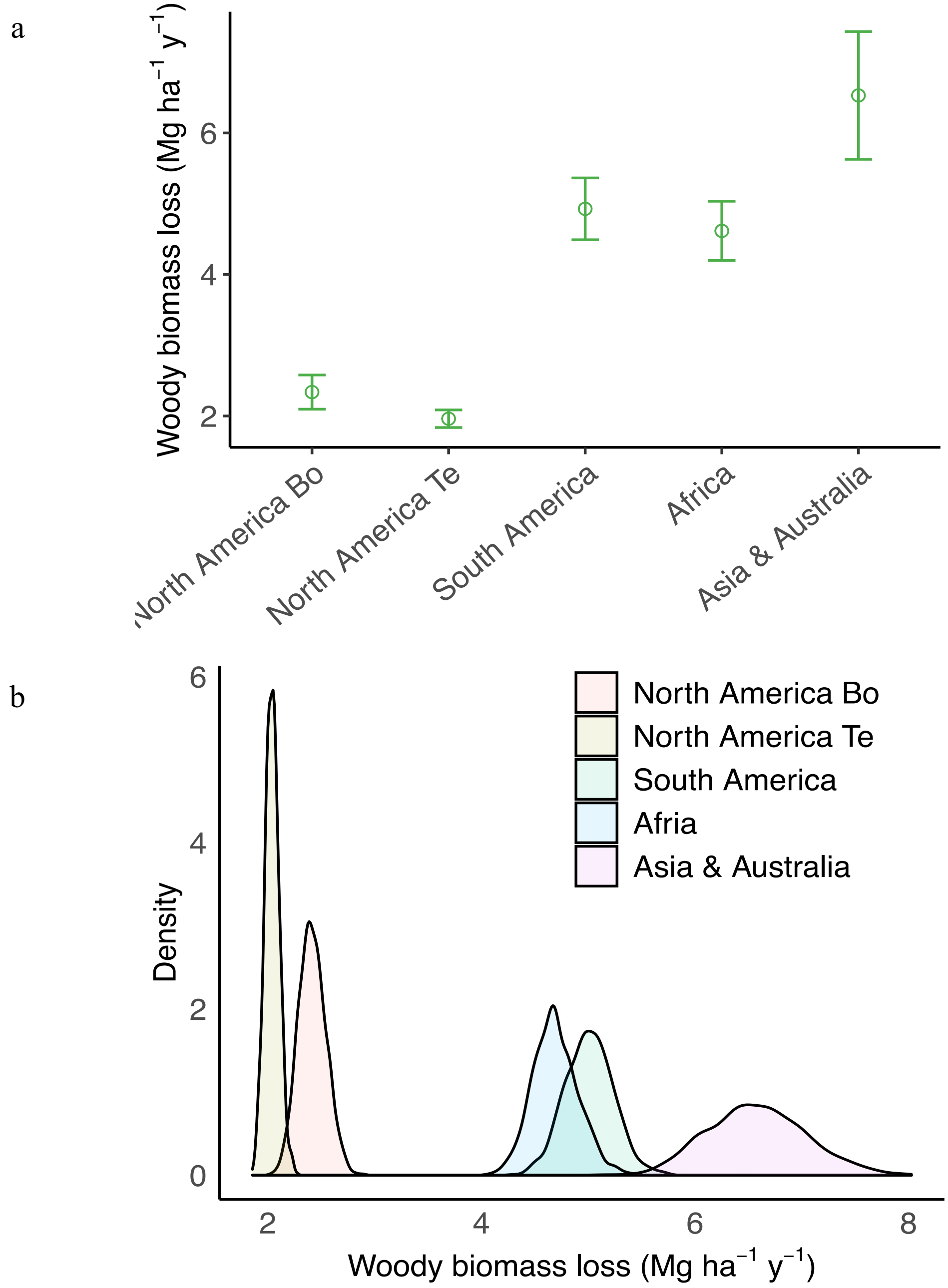

Supplementary Fig. 5. Woody biomass loss from mortality (a, LOSS, mean ± 95% CIs) and the probability distribution of the mean value of LOSS (b) across boreal and temperate forests in North America, South America, Africa, Asia & Australia. The error bars denote the 95% confidence interval.

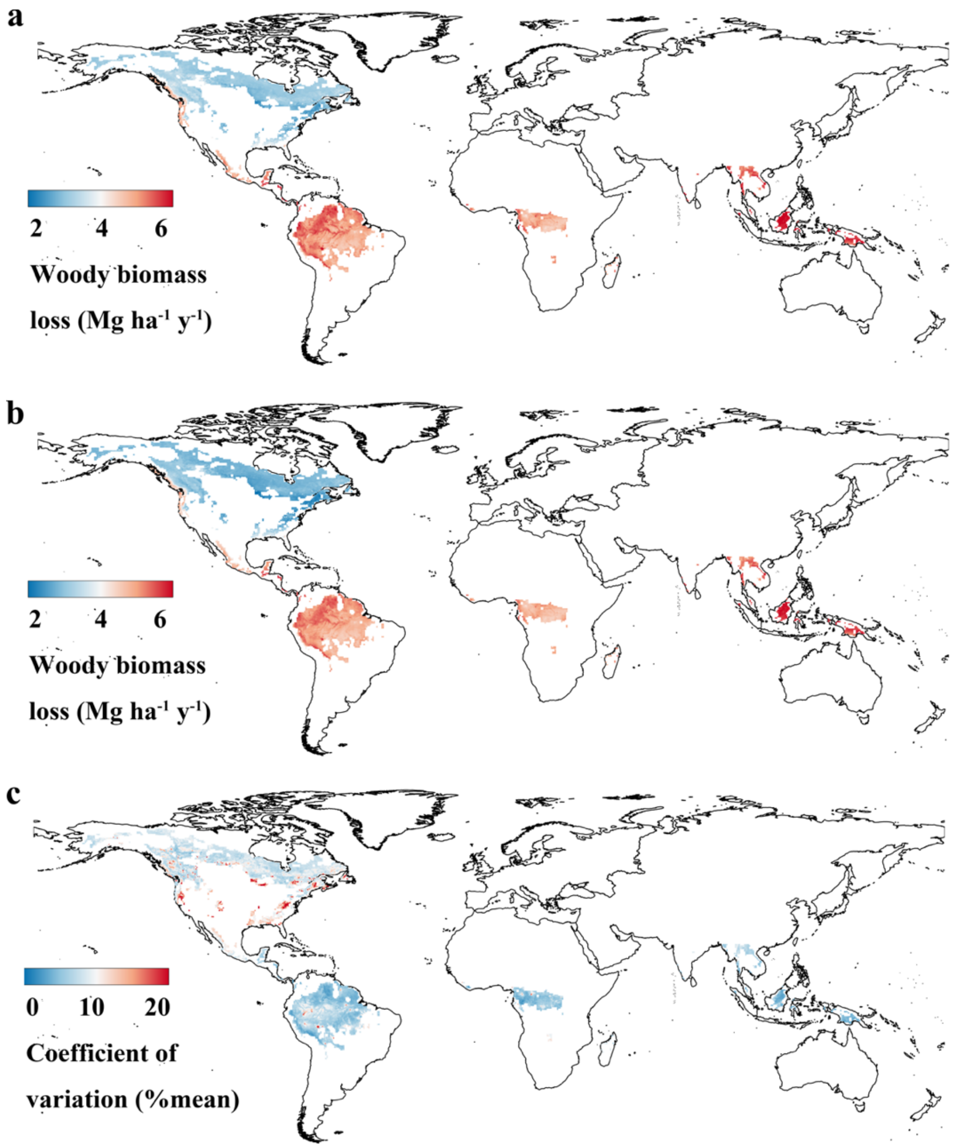

Supplementary Fig. 6. LOSS (a) across continents at 0.25 degree using full forest plot datasets and ensemble mean of LOSS (b) and its uncertainty (coefficient of variation, c) across continents at 0.25 degree derived from full forest plot data using the bootstrapped (100 iterations) approach on the sampled biomes of each point feature (LOSS) with the total number collection of LOSS points. Maps of LOSS were generated by a machine learning (random forest; see Methods for details) approach that linked spatial variations of LOSS at local-plot scale in a quasi-steady state with 57 environmental variables.

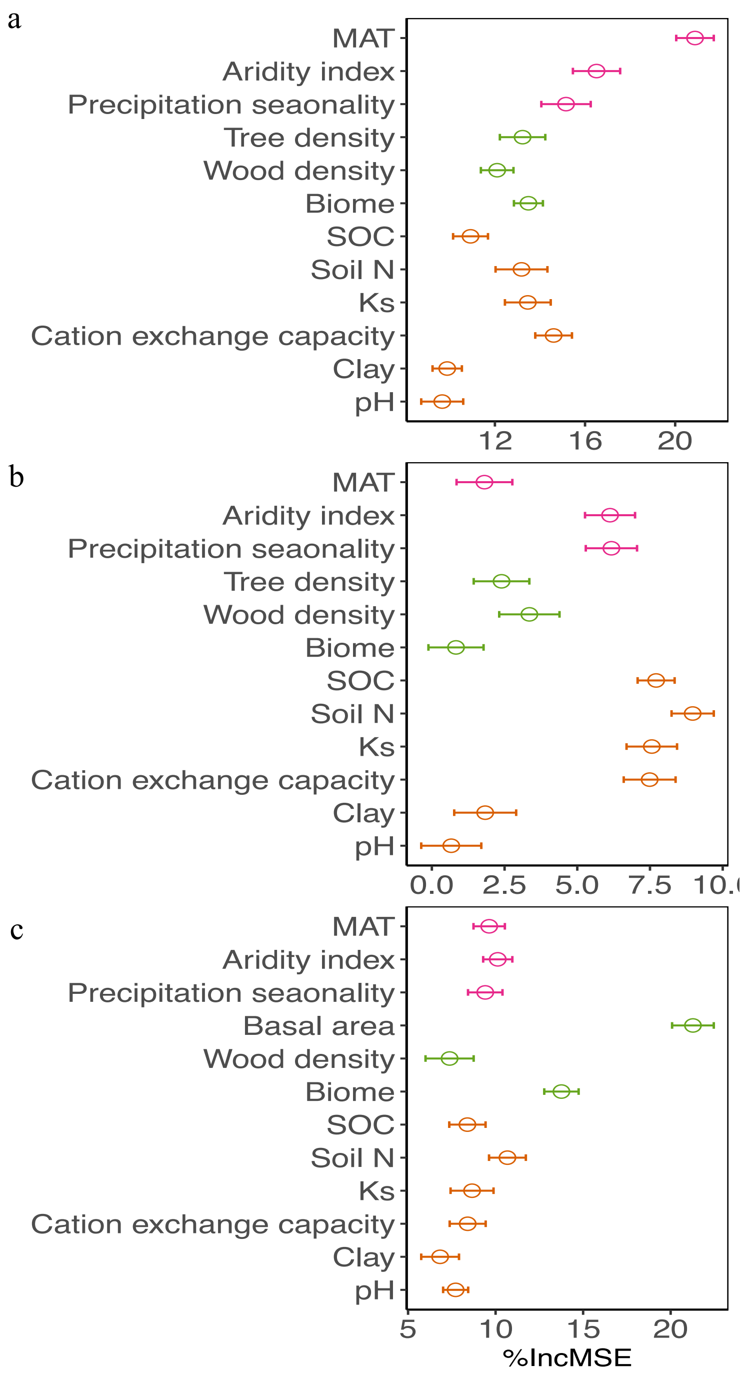

Supplementary Fig. 7. Mean decrease in accuracy (%IncMSE, mean and SD) estimated from 1000 simulations of random forests across continents (a), tropical regions (b) vs non-tropical regions (c). This is used to evaluate the importance of top environmental drivers on LOSS aggregated at 0.25 degree. The error bars denote the 95% confidence interval.

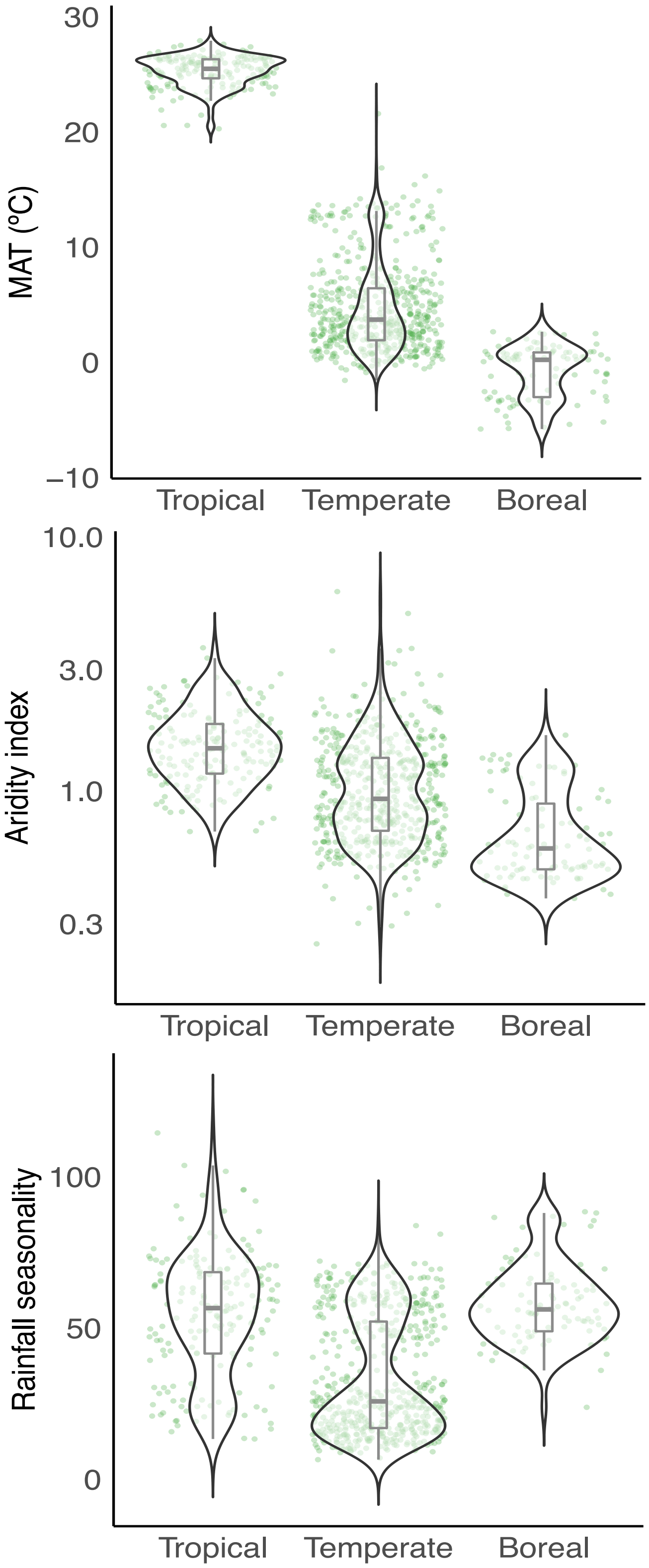

Supplementary Fig. 8. Climate gradient across tropical, temperate and boreal forests.

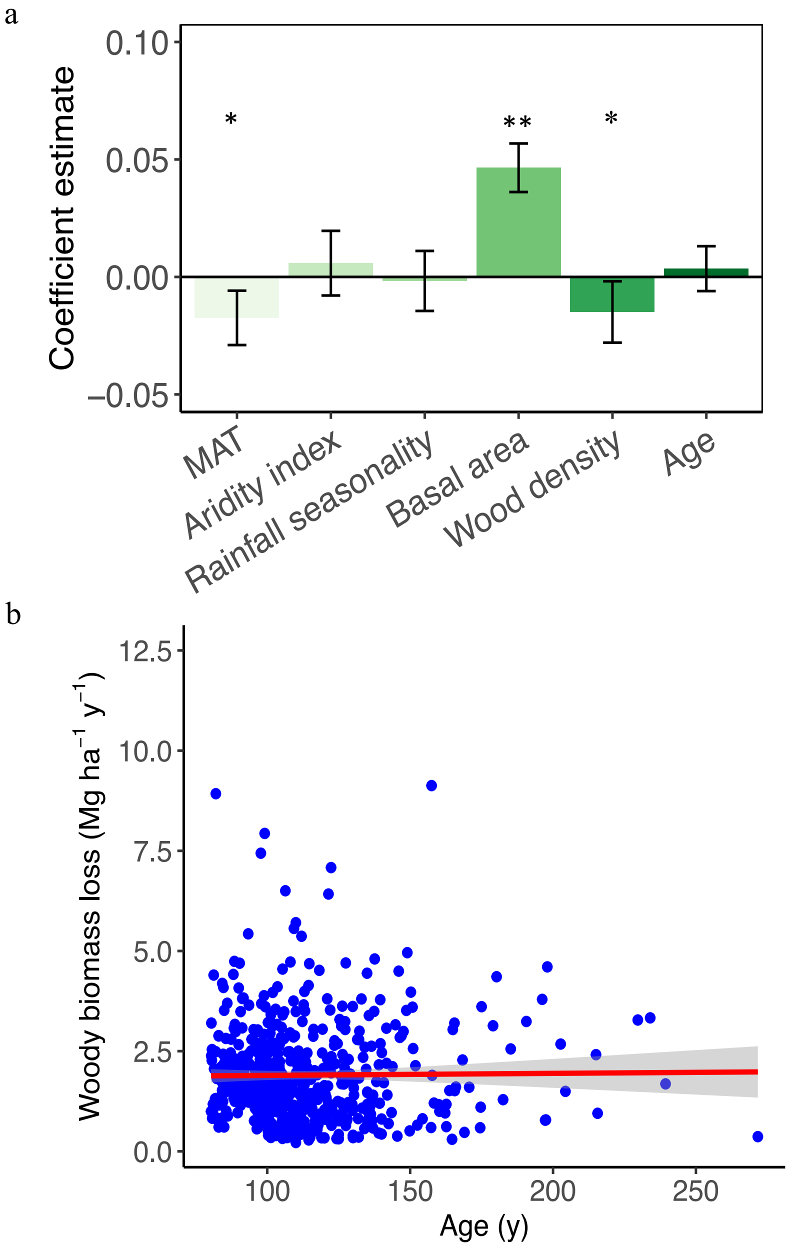

Supplementary Fig. 9. Standardized response coefficients (mean ± 95% CIs) between LOSS and dominant environmental drivers including forest age quantified by linear mixed model (a) and the linear simple regression between LOSS and forest age (b) in non-tropic regions - North America. The error bars denote the 95% confidence interval. * $P < 0.05$; ** $P < 0.01$; *** $P < 0.001$.

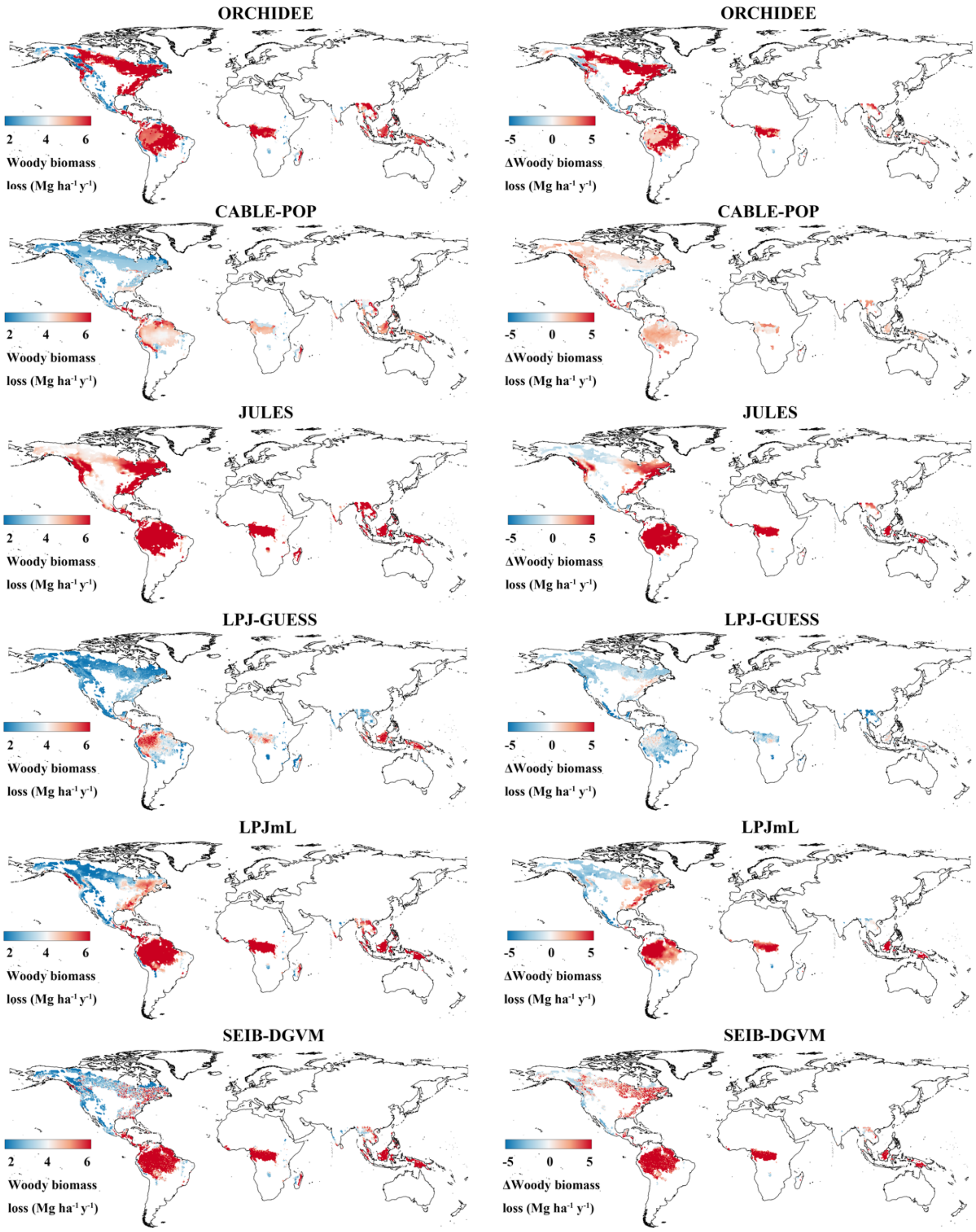

Supplementary Fig. 10. LOSS predicted in six DGVMs and the difference of LOSS between DGVMs and observational map derived from forest plot datasets using Random Forests. The LOSS map used is from Fig. 2 in the main text.

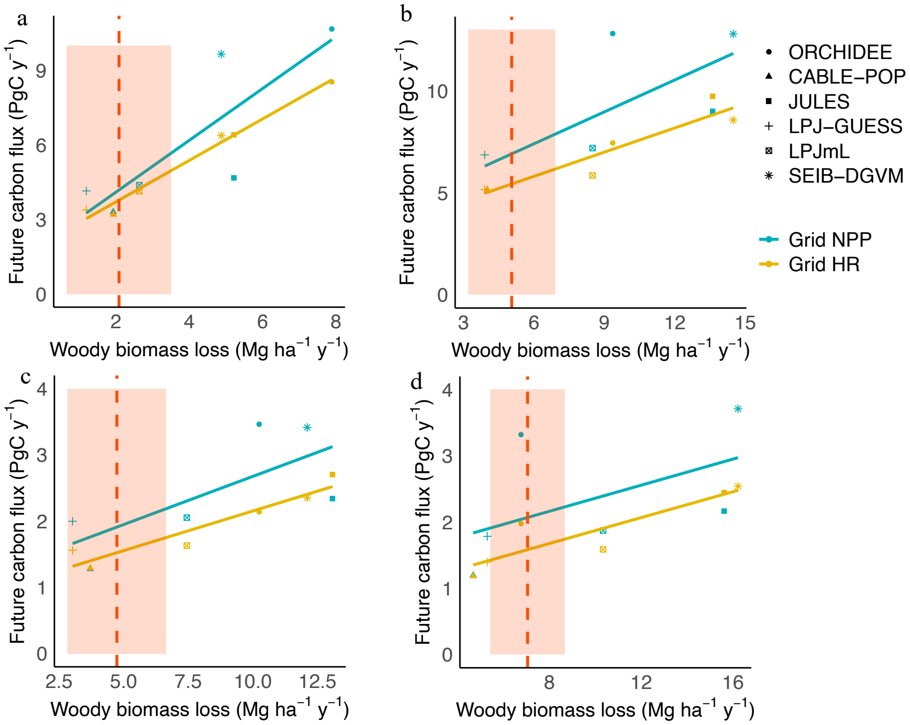

Supplementary Fig. 11. Relationship between the historic (1961-2014) LOSS at local forest plot scale and projected (2015-2099) grid-level NPP and grid-level HR (a) at continent scale (average for LOSS and sum for grid-level NPP and grid-level HR) in North America (a), South America (b), Africa (c) and Asia & Australia (d). Historic LOSS at local forest plot scale refers to the case in which LOSS was derived from DGVM outputs based on the forest plot coordinates. The data used to impose the constraint of NPP and HR were derived from local forest plot original data (mean ± sd) within each continent. Results of statistic fitting: North America: grid-level NPP – slope (1), R^2 (0.68), P (0.04); grid-level HR – slope (0.8), R^2 (0.97), P (0.0001). South America: grid-level NPP – slope (0.5), R^2 (0.54), P (0.1); grid-level HR – slope (0.4), R^2 (0.88), P (0.006). Africa: grid-level NPP – slope (0.2), R^2 (0.52), P (0.11); grid-level HR – slope (0.1), R^2 (0.89), P (0.004). Asia & Australia: grid-level NPP – slope (0.1), R^2 (0.27), P (0.29); grid-level HR – slope (0.1), R^2 (0.8), P (0.016).

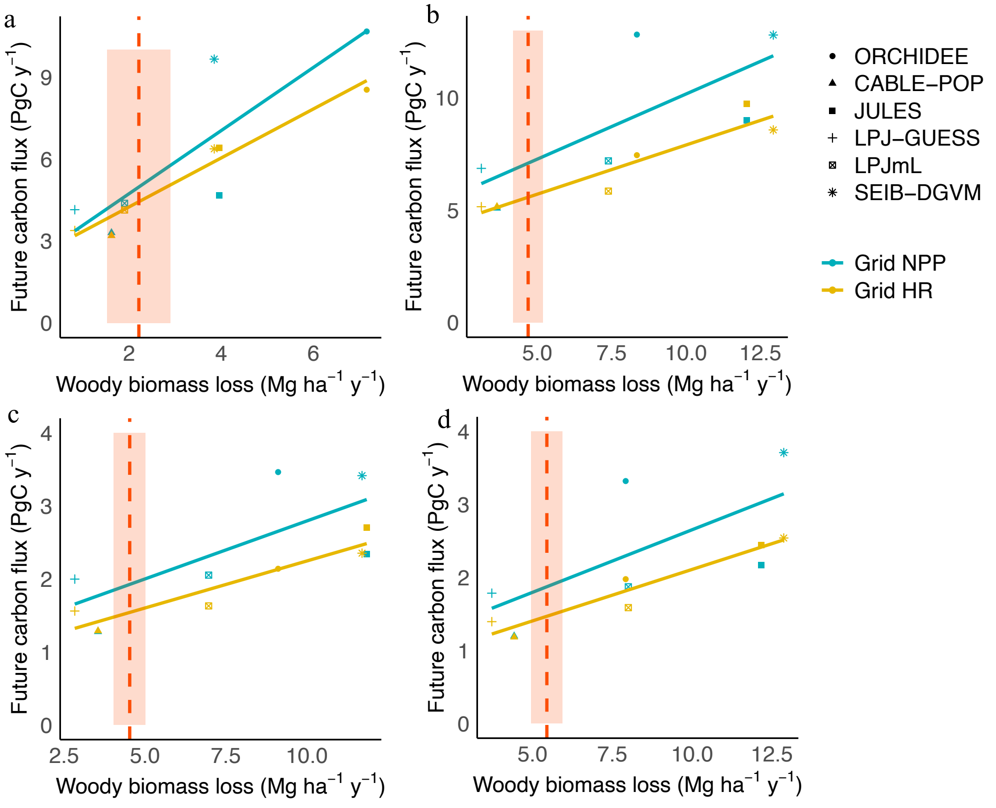

Supplementary Fig. 12. Relationship between the historic (1961-2014) LOSS at continent scale and projected (2015-2099) grid-level NPP and grid-level HR (a) at continent scale (average for LOSS and sum for grid-level NPP and grid-level HR) in North America (a), South America (b), Africa (c) and Asia & Australia (d). Historic LOSS at continent scale refers to the case in which LOSS was derived from DGVM outputs based on the coordinates of map of LOSS. The data used to impose the constraint of NPP and HR were derived from map of LOSS (mean ± sd) within each continent. Results of statistic fitting: North America: grid-level NPP – slope (1.2), R^2 (0.7), P (0.04); grid-level HR – slope (0.9), R^2 (0.95), P (0.0008). South America: grid-level NPP – slope (0.6), R^2 (0.54), P (0.1); grid-level HR – slope (0.4), R^2 (0.88), P (0.006). Africa: grid-level NPP – slope (0.2), R^2 (0.52), P (0.11); grid-level HR – slope (0.1), R^2 (0.87), P (0.006). Asia & Australia: grid-level NPP – slope (0.2), R^2 (0.45), P (0.15); grid-level HR – slope (0.1), R^2 (0.91), P (0.003). The LOSS map used during the constraint is from Fig. 2 in the main text.

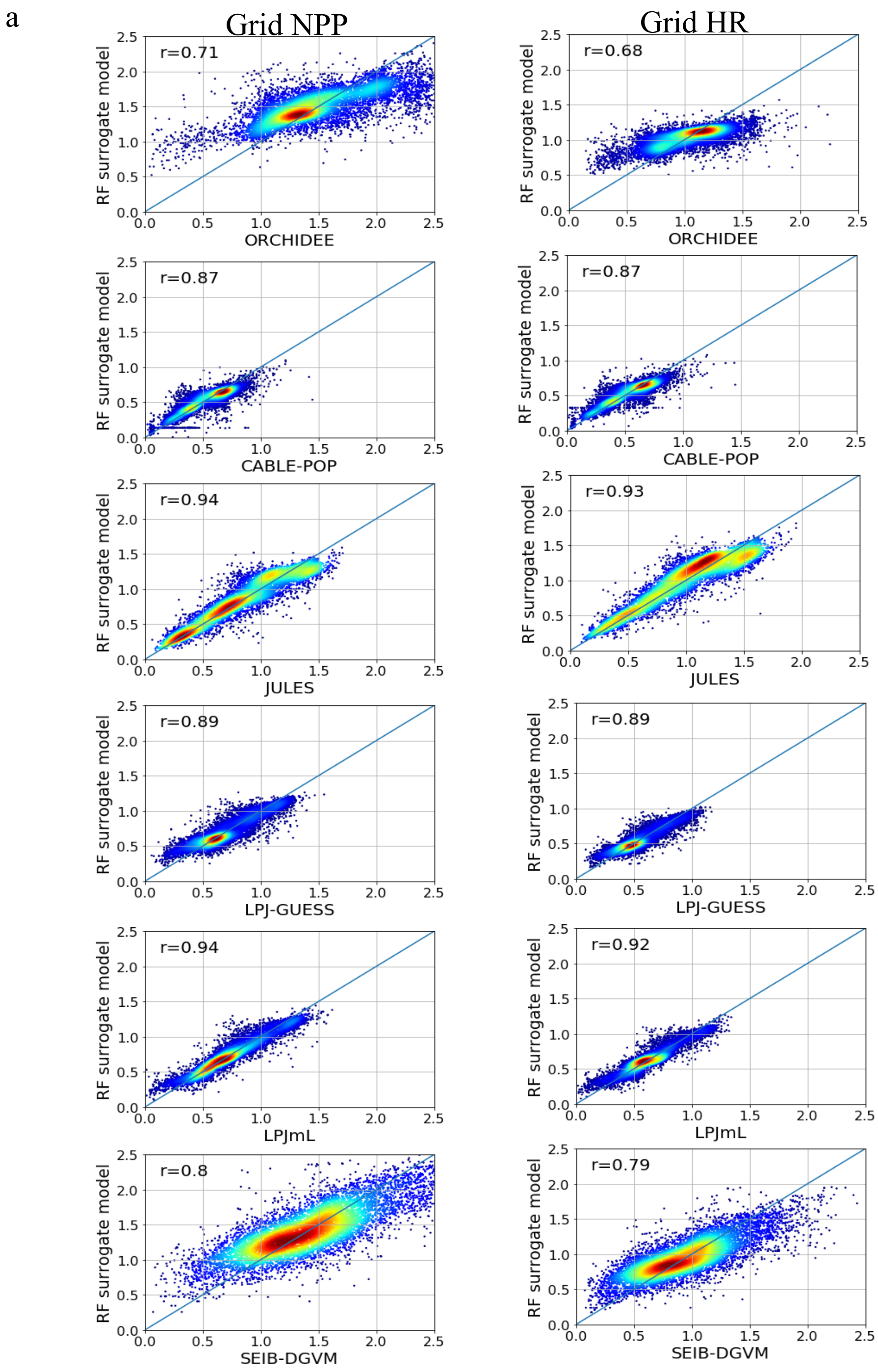

a
Grid NPP
Grid HR
RF surrogate model
r=0.71
ORCHIDEE
r=0.68
ORCHIDEE
r=0.87
CABLE-POP
r=0.87
CABLE-POP
r=0.94
JULES
r=0.93
JULES
r=0.89
LPJ-GUESS
r=0.89
LPJ-GUESS
r=0.94
LPJmL
r=0.92
LPJmL
r=0.8
SEIB-DGVM
r=0.79
SEIB-DGVM

b

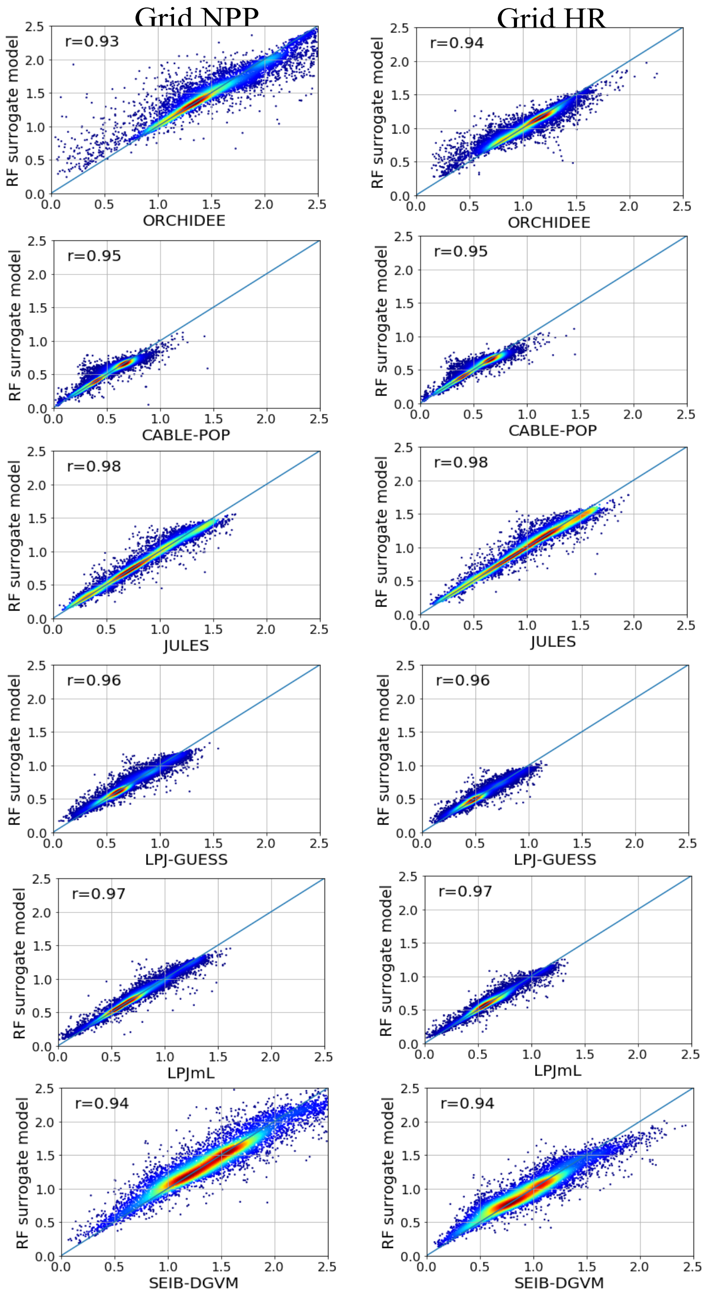

Supplementary Fig. 13. The relationships between projected (2015-2099) grid-level NPP and grid-level HR estimated in DGVMs (ORCHIDEE, CABLE-POP, JULES, LPJ-GUESS, LPJmL, and SEIB-DGVM) and a machine learning version of DGVM that surrogates existing processes by building the non-linear relationships between historical LOSS and projected NPP and HR without (a) and with climate effects (b) (see Methods).

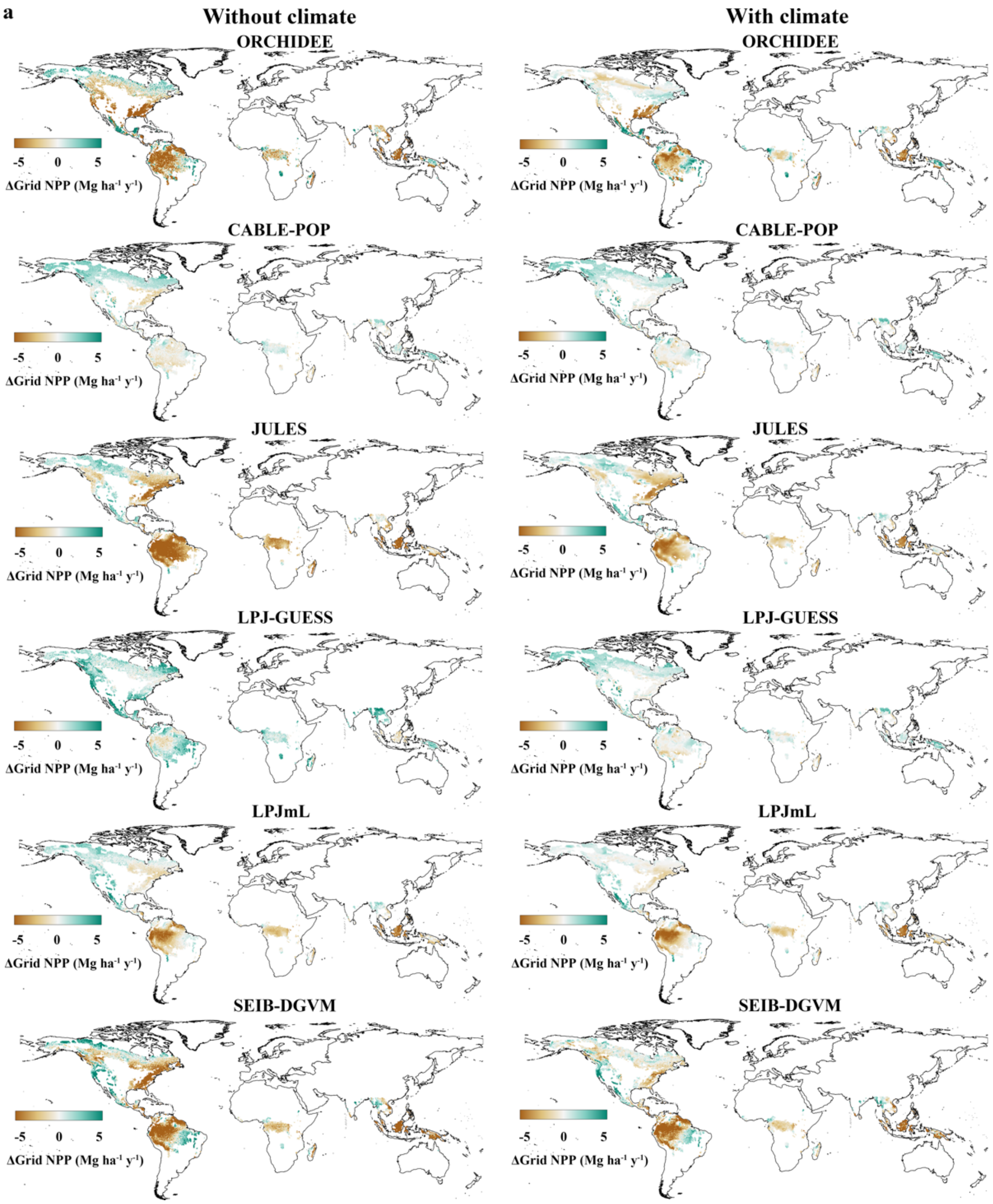

a
Without climate
With climate
ORCHIDEE
CABLE-POP
JULES
LPJ-GUESS
LPJmL
SEIB-DGVM
-5
0
5
ΔGrid NPP (Mg ha-1 y-1)

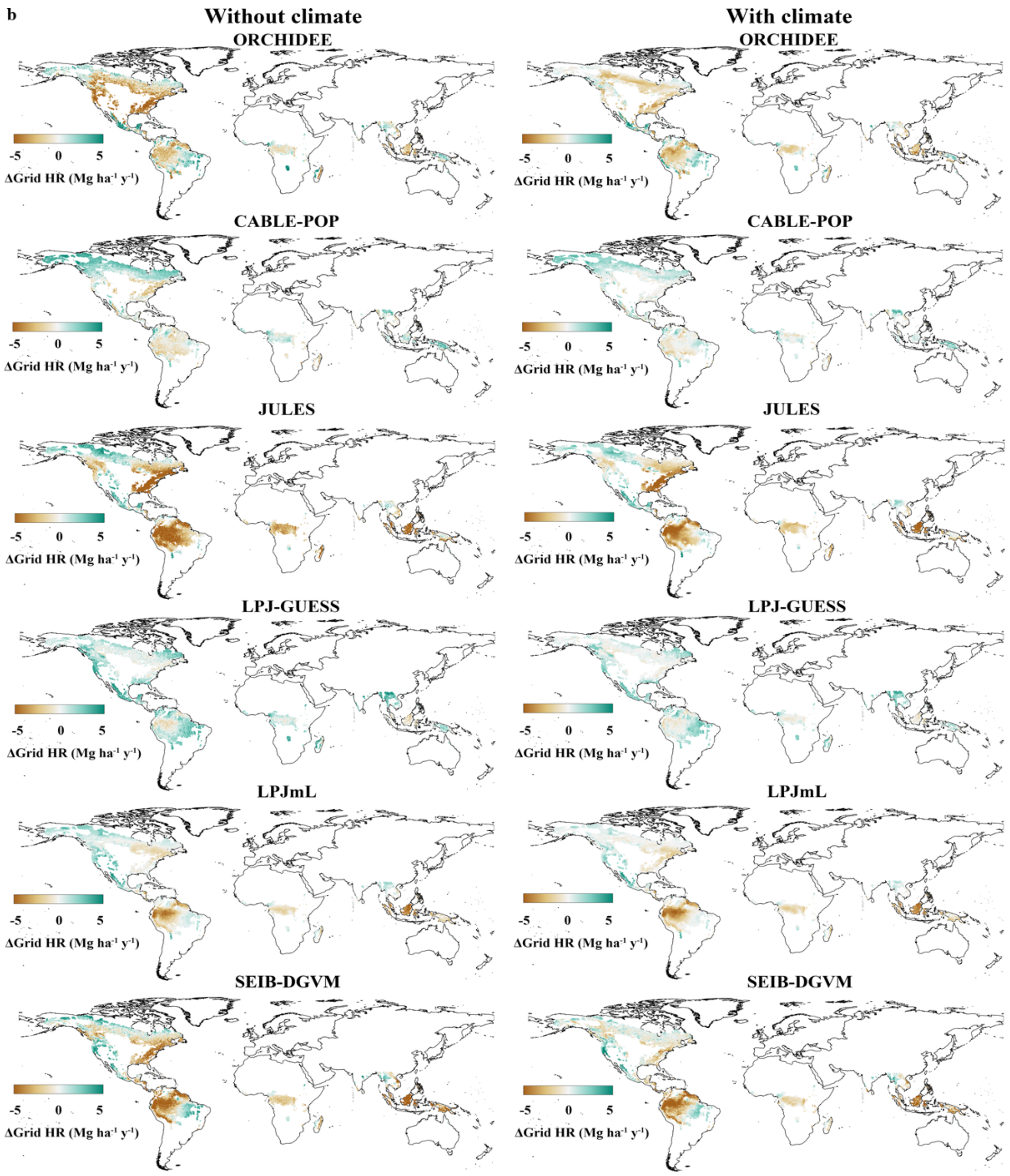

Supplementary Fig. 14. The difference of projected (2015-2099) grid-level NPP (a) and grid-level HR (b) after constraint and before constraint. The constraint was achieved by using the ML algorithm to build the non-linear relationships without and with climate effects between historical LOSS (1961-2014) and projected grid-level NPP and grid-level HR in DGVMs. The ML algorithm was then forced by the observational LOSS to constrain the projected grid-level NPP and grid-level HR. The LOSS map used for constraint is from Fig. 2 in main text.

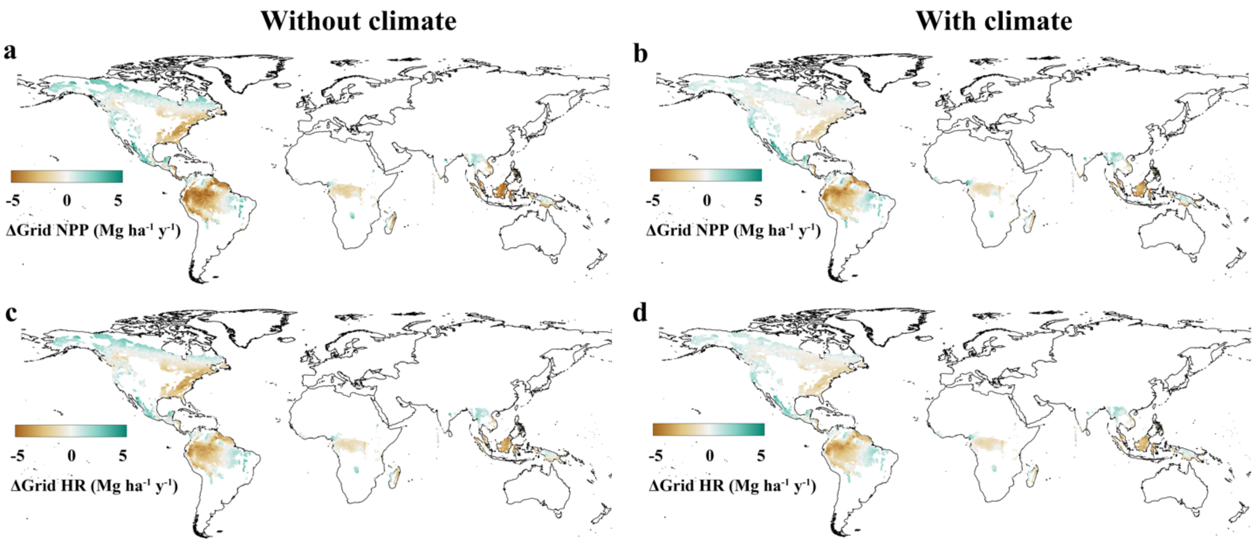

Supplementary Fig. 15. Ensemble mean of the difference of projected (2015-2099) grid-level NPP (a, b) and grid-level HR (c, d) after constrain without and with climate effects and before constrain in DGVMs. The LOSS map used for constraint is from Fig. 2 in main text.

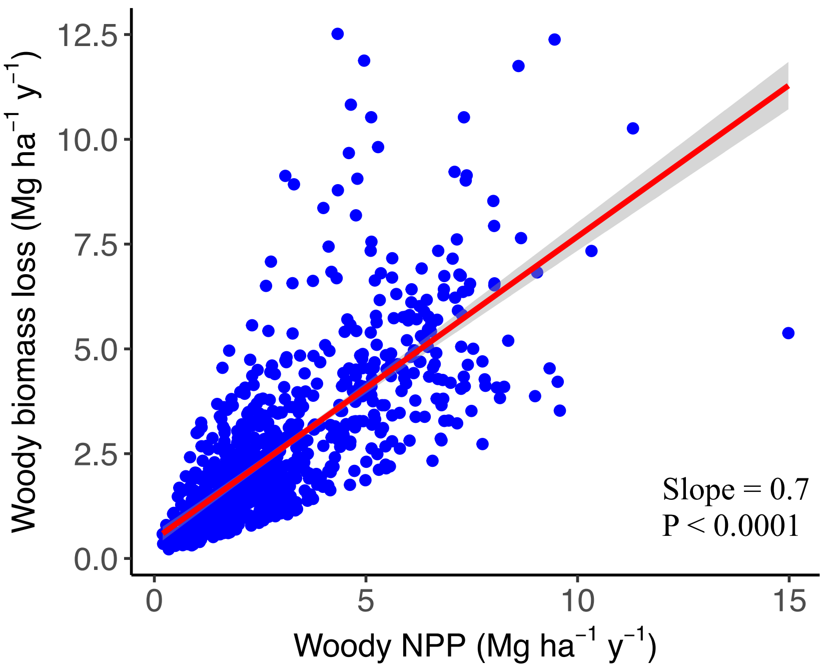

Supplementary Fig. 16. The relationships between growth – woody NPP and woody biomass loss from mortality derived from forest plot datasets across continents.

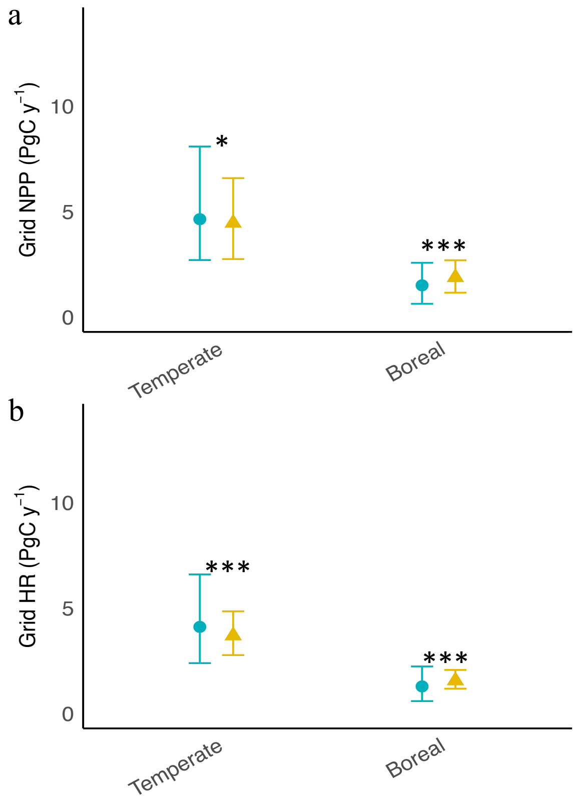

Supplementary Fig. 17. Projected grid-level NPP and grid heterotrophic respiration (HR) in temperate and boreal forests in North America. (a, b) Projected (2015-2099) grid-level NPP (a) and grid-level HR (b) in temperate and boreal forests quantified by six dynamic vegetation models - DGVMs (ORCHIDEE, CABLE-POP, JULES, LPJ-GUESS, LPJmL, and SEIB-DGVM). The y axes are the minimum, mean, and maximum values in six DGVMs. 'DGVMs' refers to the scenario before constraint and 'DGVMs + Observation' refers to the scenario after constraint without climate predictors. The constraint was achieved by using the observational maps (n = 100; through a bootstrapping approach; see Methods for details) of biomass loss to mortality (LOSS) derived from forest plots data to feed into the trained ML (random forest). model to assess the impacts of historic LOSS on the projected NPP and HR in each DGVM. Reported are ensemble means of constraint.

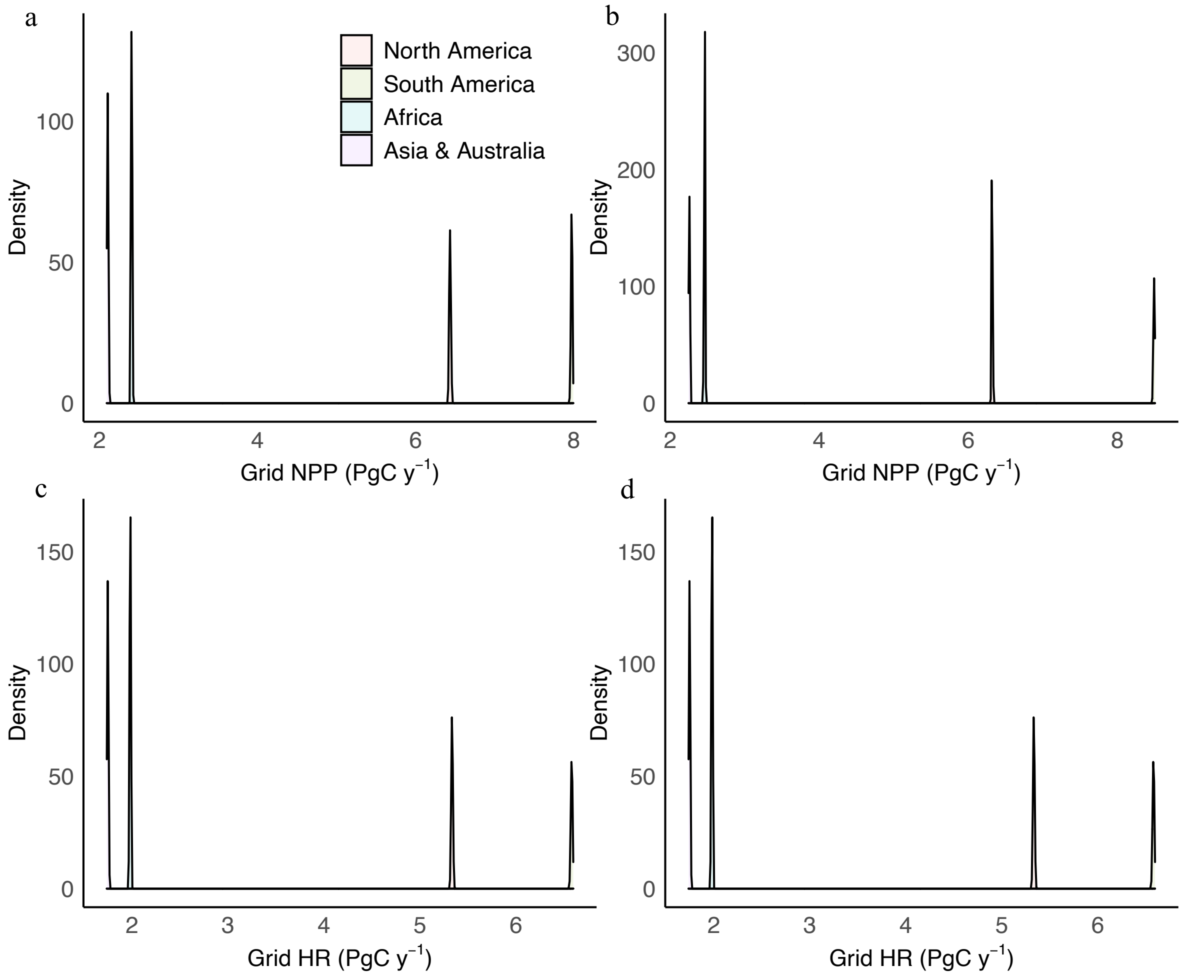

Supplementary Fig. 18. The probability distribution of projected NPP (a, b) and HR (c, d) after constraint without climate (a, c) and with climate (b, d) effects in the bootstrapping (100 times) approach (see Methods for details).

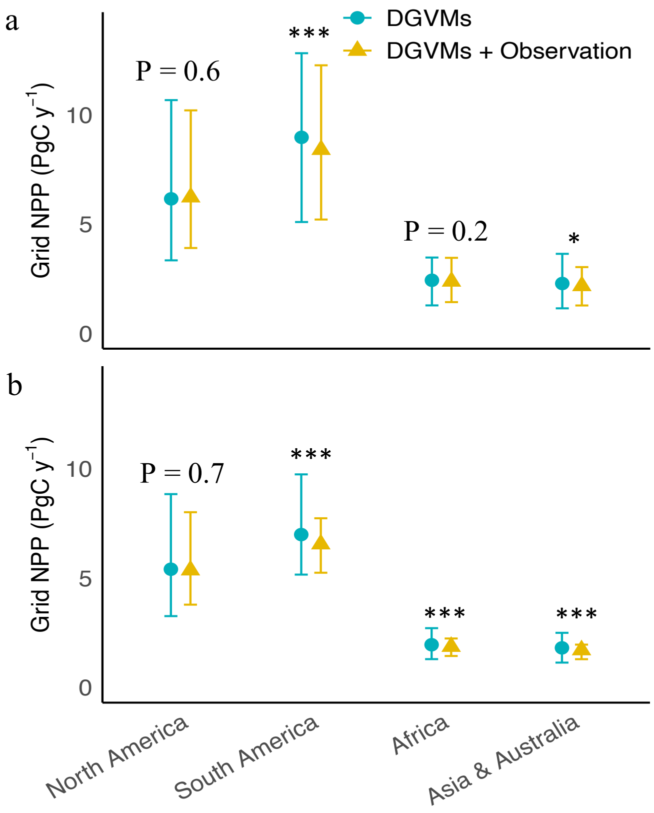

Supplementary Fig. 19. Projected (2015-2099) grid-level NPP (a) and grid-level HR (b) across continents quantified by six dynamic vegetation models - DGVMs (ORCHIDEE, CABLE-POP, JULES, LPJ-GUESS, LPJmL, and SEIB-DGVM). The y axes are the minimum, mean, and maximum values in six DGVMs. 'DGVMs' refers to the scenario before constraint and 'DGVMs + Observation' refers to the scenario after constraint with climate (precipitation and temperature) predictors. The constraint was achieved by using the observational maps (n = 100; through a bootstrapping approach; see Methods for details) of biomass loss to mortality (LOSS) derived from forest plots data to feed into the trained ML (random forest) model to assess the impacts of historic LOSS on the projected NPP and HR in each DGVM. Reported are ensemble means of constraint.

a

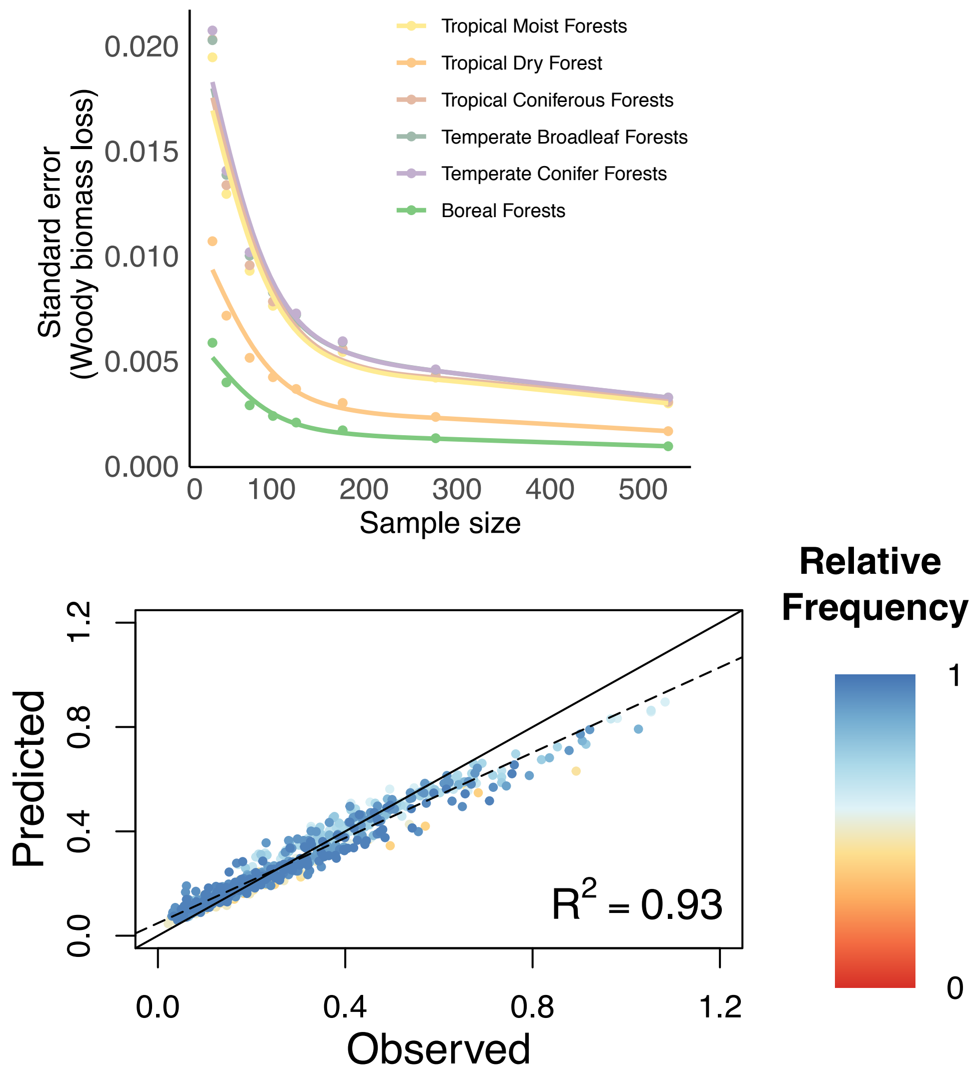

Supplementary Fig. 20. The standard error of the predicted mean values of LOSS (a) decrease with increasing sample size, quantified by the 1000 bootstrapping. The relationships (b) between predicted versus observed values of LOSS for the best final models using heat plots. Dashed diagonal lines indicate fitted relationships, while solid diagonal lines indicate a 1:1 relationship between predicted and observed points.

Supplementary References

1. Luo, Y. & Chen, H. Y. H. Observations from old forests underestimate climate change effects on tree mortality. *Nat. Commun.* **4**, (2013).
2. Luo, Y. & Chen, H. Y. H. Climate change-associated tree mortality increases without decreasing water availability. *Ecology Letters* vol. 18 1207–1215 (2015).
3. Chen, H. Y. H., Luo, Y., Reich, P. B., Searle, E. B. & Biswas, S. R. Climate change-associated trends in net biomass change are age dependent in western boreal forests of Canada. *Ecology letters* vol. 19 1150–1158 (2016).
4. Pugh, T. A. M. *et al.* Role of forest regrowth in global carbon sink dynamics. *Proc. Natl. Acad. Sci.* **116**, 4382–4387 (2019).
5. Zhu, K., Zhang, J., Niu, S., Chu, C. & Luo, Y. Limits to growth of forest biomass carbon sink under climate change. *Nature Communications* (2018).
6. Yu, K. *et al.* Pervasive decreases in living vegetation carbon turnover time across forest climate zones. *Proc. Natl. Acad. Sci. U. S. A.* (2019) doi:10.1073/pnas.1821387116.
7. Trugman, A. T., Medvigy, D., Anderegg, W. R. L. & Pacala, S. W. Differential declines in Alaskan boreal forest vitality related to climate and competition. *Glob. Chang. Biol.* (2017) doi:10.1111/gcb.13952.
8. Brienen, R. J. W. *et al.* Long-term decline of the Amazon carbon sink. *Nature* **519**, 344–348 (2015).
9. Hubau, W. *et al.* Asynchronous carbon sink saturation in African and Amazonian tropical forests. *Nature* **579**, (2020).
10. Sullivan, M. J. P. *et al.* Long-term thermal sensitivity of earth’s tropical forests. *Science (80-.).* **368**, (2020).

11. Anderson-Teixeira, K. J. *et al.* ForC: a global database of forest carbon stocks and fluxes. *Ecology* vol. 99 (2018).

12. Hengl, T., Nussbaum, M., Wright, M. N., Heuvelink, G. B. M. & Gräler, B. Random forest as a generic framework for predictive modeling of spatial and spatio-temporal variables. *PeerJ* **6**, e5518 (2018).

13. DeFries, R., Hansen, M., Townshend, J. R. G., Janetos, A. C. & Loveland, T. R. 1 Kilometer Tree Cover Continuous Fields, 1.0. *Dep. Geogr. Univ. Maryland, Coll. Park. Maryl.* (2000).

14. Tuanmu, M. N. & Jetz, W. A global 1-km consensus land-cover product for biodiversity and ecosystem modelling. *Glob. Ecol. Biogeogr.* **23**, 1031–1045 (2014).

15. Liu, Y. *et al.* Field-experiment constraints on the enhancement of the terrestrial carbon sink by CO2 fertilization. *Nat. Geosci.* **12**, (2019).

16. Cox, P. M. *et al.* Sensitivity of tropical carbon to climate change constrained by carbon dioxide variability. *Nature* **494**, (2013).
