# Supplementary material for: Field-based tree mortality constraint reduces estimates of model-projected forest carbon sinks
Source: Nat Commun. 2022 Apr 19;13:2094. doi: 10.1038/s41467-022-29619-4 (PMC9018757; doi:10.1038/s41467-022-29619-4)
Supplement: Supplementary file 3 — Description of Additional Supplementary Files [file 41467_2022_29619_MOESM3_ESM.pdf]

Description of Additional Supplementary Files

File Name: Supplementary Data 1
Description: A list of 58 environmental variables used to generate the spatially explicit map of LOSS using the machine learning approach (random forest). Please refers to the attached excel sheet.
